# Exploration of a diversity of computational and statistical measures of association for genome-wide genetic studies

**DOI:** 10.1186/s13040-019-0201-4

**Published:** 2019-07-09

**Authors:** Elisabetta Manduchi, Patryk R. Orzechowski, Marylyn D. Ritchie, Jason H. Moore

**Affiliations:** 1 Institute for Biomedical Informatics, University of Pennsylvania, Philadelphia, PA USA; 2 Department of Biostatistics, Epidemiology and Informatics, University of Pennsylvania, Philadelphia, PA USA; 3 Department of Genetics, University of Pennsylvania, Philadelphia, PA USA

**Keywords:** GWAS, Association analysis, Univariate analysis, Ranked list, Canberra metric

## Abstract

**Background:**

The principal line of investigation in Genome Wide Association Studies (GWAS) is the identification of main effects, that is individual Single Nucleotide Polymorphisms (SNPs) which are associated with the trait of interest, independent of other factors. A variety of methods have been proposed to this end, mostly statistical in nature and differing in assumptions and type of model employed. Moreover, for a given model, there may be multiple choices for the SNP genotype encoding. As an alternative to statistical methods, machine learning methods are often applicable. Typically, for a given GWAS, a single approach is selected and utilized to identify potential SNPs of interest. Even when multiple GWAS are combined through meta-analyses within a consortium, each GWAS is typically analyzed with a single approach and the resulting summary statistics are then utilized in meta-analyses.

**Results:**

In this work we use as case studies a Type 2 Diabetes (T2D) and a breast cancer GWAS to explore a diversity of applicable approaches spanning different methods and encoding choices. We assess similarity of these approaches based on the derived ranked lists of SNPs and, for each GWAS, we identify a subset of representative approaches that we use as an ensemble to derive a union list of top SNPs. Among these are SNPs which are identified by multiple approaches as well as several SNPs identified by only one or a few of the less frequently used approaches. The latter include SNPs from established loci and SNPs which have other supporting lines of evidence in terms of their potential relevance to the traits.

**Conclusions:**

Not every main effect analysis method is suitable for every GWAS, but for each GWAS there are typically multiple applicable methods and encoding options. We suggest a workflow for a single GWAS, extensible to multiple GWAS from consortia, where representative approaches are selected among a pool of suitable options, to yield a more comprehensive set of SNPs, potentially including SNPs that would typically be missed with the most popular analyses, but that could provide additional valuable insights for follow-up.

**Electronic supplementary material:**

The online version of this article (10.1186/s13040-019-0201-4) contains supplementary material, which is available to authorized users.

## Introduction

GWAS have yielded many valuable insights into the genetic bases of common diseases and complex traits. Several reviews over the years have discussed theoretical and practical considerations as well as achievements relative to this type of studies [1–4]. The large majority of human GWAS efforts to date have focused on detecting main effects, i.e. individual SNPs that are associated with a given complex trait, based on the assumption that most SNPs contribute to a given trait in an additive manner [5], independently of genetic background and environmental exposure. Several statistical methods have been proposed for univariate GWAS analyses, as reviewed in [6]. Some of these require a choice of genotype encoding and the prevailing choice is additive encoding, where the genotype of an individual at a SNP is represented by 0, 1, or 2 to indicate the number of non-reference alleles. Other possible encodings are the dominant encoding, where the homozygous referent genotype is encoded with 0 and the other genotypes with 1, and the recessive encoding, where the homozygous alternate genotype is encoded with 1 and the other genotypes with 0. Besides statistical methods, machine learning methods are also a possibility. Machine learning has not been explored for univariate associations, as most use it for interactions or other complex effects, but methods such as MDR [7], entropy-based measures [8], and decision trees [9] could also be employed to detect main effects.

In the context of a single GWAS or of multiple GWAS for the same trait within a consortium, the standard practice is to make a specific choice of main effect method and (when applicable) genetic encoding. But typically multiple methods are applicable to a given GWAS and different methods and encodings may uncover different facets of the genetic mechanisms underlying a trait. Given the availability of several software packages implementing these approaches, it is relatively straightforward to employ multiple approaches to potentially expand the set of candidate SNPs for follow-up studies. In this work we focus on binary trait GWAS and, using as case studies a Type 2 Diabetes (T2D) and a breast cancer GWAS, we explore 25 applicable approaches spanning different methods, and their software implementations, as well as encoding choices. For each GWAS, we assess similarity of these approaches using Canberra-based distances of the ranked lists thereby derived. Based on this, we identify a representative collection of approaches that we use as an ensemble to derive a union list of top SNPs for each GWAS. Among these are SNPs identified by multiple approaches as well as several SNPs identified by only one or a few of the less frequently used approaches. The latter include SNPs from established loci and SNPs which have other supporting lines of evidence in terms of their potential relevance to the traits. These would typically be missed with the most popular analyses, yet they could provide additional valuable insights for follow-up. Based on this we propose a multi-approach workflow in main effect analyses, where the choice of suitable methods depends on the GWAS being analyzed and the assumptions that can be made. Such a workflow is applicable also in the case of multiple data sets for the same trait within a consortium and could be utilized, mutatis mutandis, also in the quantitative trait scenario.

## Methods

### GWAS data sets

The GWAS data sets used for this work are available, upon application, from the database of Genotypes and Phenotypes (dbGaP; [10]) under the indicated accessions. These are:

- The GENEVA Genes and Environment Initiatives in T2D, available under phs000091.v2.p1 (https://www.ncbi.nlm.nih.gov/projects/gap/cgi-bin/study.cgi?study_id=phs000091.v2.p1).
- The breast cancer component of the Breast and Prostate Cancer Cohort Consortium (BPC3) GWAS of Aggressive Prostate Cancer and ER- Breast Cancer, available under phs000812.v1.p1 (https://www.ncbi.nlm.nih.gov/projects/gap/cgi-bin/study.cgi?study_id=phs000812.v1.p1).

### GWAS pre-processing

We used PLINK v1.9 (https://www.cog-genomics.org/plink2/), bcftools (http://www.sanger.ac.uk/science/tools/samtools-bcftools-htslib) and vcftools [11] to manipulate and filter these data sets. For both data sets our starting points were the plink files made available from dbGaP. In the case of GENEVA, we started from the dbGaP ‘zero-out’ plink files (where a set of specific SNPs in specific samples had been set to 0, because a chromosome anomaly or quality problem was detected) and merged the NHS and HPFS data after removal of the duplicate markers and duplicated/related individuals annotated in the provided sample files. We mapped coordinates to hg19 and then applied the following filters in the listed order:

1. Individuals failing the PLINK ‘--check-sex’ were removed.
2. Markers with missing-call rate exceeding 0.01 were removed.
3. Individuals with missing-call rate exceeding 0.01 were removed.
4. Markers with Minor Allele Frequency (MAF) below 0.05 were removed.
5. Markers with Hardy-Weinberg equilibrium exact test *p*-value below 0.00001 were removed.
6. Individuals were filtered based on relatedness according to steps 11–13 of [12], but with a threshold of 0.125 (instead of 0.185) for IBS. This only removed 29 individuals in the GENEVA data set and 8 individuals in the BPC3 data set.
7. Steps 2–5 where then repeated.
8. Markers with different genotype call rates between cases and controls according to steps 24–25 of [12] were removed.

We used the +fixref plugin in bcftools to fix strand issues. Principal Components (PCs) were obtained using the PLINK –pca command after Linkage Disequilibrium (LD) pruning (−indep-pairwise 50 5 0.2). We considered autosomal biallelic markers only in the analyses described below. Moreover, for GENEVA we only retained individuals whose race was annotated as ‘white’ in the sample information file. Table 1 summarizes the number of individuals and markers in each data set after this pre-processing.

### Univariate analysis approaches

We carried out univariate analyses for each data set using seven different software packages corresponding to methods applicable to the binary trait GWAS utilized in this work. Since some of the packages offered different options controlling either the method or the genetic encoding employed, in total we applied 25 different approaches, where by ‘approach’ we refer to a choice of method, encoding, and implementation. After assessing association of available covariates with the trait in each data set, we adjusted for age in GENEVA and for age and PC1-PC6 in BPC3 for each method that allowed covariate adjustment. Table 2 summarizes the 25 approaches with information on whether covariate adjustment was available and information on the final output used to rank SNPs.

#### Statistical approaches

We refer to [6] for a general review covering most of the statistical methods we employed. Here we indicate specific references for each method implementation and notes on how we used them.

- For the PLINK analyses we used v1.9 indicated above.
- The logistic regression analyses implemented in PLATO [13], allow for an additional type of encoding, namely the codominant encoding. In the latter, each marker uses two variables as a dummy encoding of a categorical variable. The “Het” variable is 1 only when the marker is heterozygous, and the “Hom” variable is 1 only when the marker is homozygous alternate (see https://ritchielab.org/files/RL_software/plato-manual-2.1.pdf).
- CARAT [14] performs genome-wide association analysis of a binary trait in individuals possibly subject to unobserved population structure and is based on a retrospective mixed-effects quasi-likelihood framework. We ran CARAT in two ways: (i) once for the whole data set, asking the software to use a Genetic Relationship Matrix (GRM) based on all markers after LD pruning; (ii) using a Leave-One-Chromosome-Out (LOCO) approach, where we ran CARAT separately for each chromosome with a GRM based on all markers not on that chromosome after LD pruning.
- LTMLM [15] employs a liability threshold-based mixed model association statistic.
- LEAP [16] uses a liability estimator approach.

#### Machine-learning approaches

We considered three approaches:

1. MDR [7], in its java implementation version 3.0.4_dev. For univariate analyses, the software is run for 1-way models only, where the model for a given SNP assigns to each genotype a high risk if the ratio of cases to controls for that genotype exceeds the overall ratio of cases to controls in the data set. The balanced accuracy is computed using 10-fold cross validation. Genotypes are input as the number of non-reference alleles, as 1-way MDR adaptively discovers the encodings.
2. Entropy [8], which yields a score for each SNP representing the reduction in the uncertainty of the phenotype due to the knowledge about the genotype (again input as number of non-reference alleles) at that SNP. The MDR java software provides a tool to compute this too.
3. Decision Trees [9] are popular classifiers which look for the splits of the given features which best predict the endpoint. For each of the SNPs, with genotype encoded as the number of non-reference alleles, we constructed a separate decision tree using scikit-learn (http://scikit-learn.org/). Thus, in this application, the decision tree simply calculates a cross-validated purity measure. Each input dataset was divided into training and testing datasets with 75 and 25% proportions. We used the following space of hyper parameters for DecisionTreeClassifier: criterion was set to either ‘gini’ or ‘entropy’ value, and ‘min_impurity_decrease’ was within the following values {0, 0.1, 0.2, 0.3, 0.4, 0.5}. In order to determine the best parameters for each decision tree, for each of the SNPs a 5-fold cross validation with r^2^ score as a quality measure was applied. The best settings obtained with this hyper-parameter tuning was then used to check how each SNP could predict the phenotype.

### Similarity assessments

To compare results from the 25 different approaches described above for each data set, we utilized Canberra-based distance metrics. More precisely, since each approach yields a ranked list of SNPs (by increasing *p*-values or by decreasing score, depending on the approach), we measured the similarity of two approaches by comparing the resulting SNP rankings using the metrics described in [17]. The Canberra distance between two different rankings σ and τ of a list of SNPs is given by the formula:

$$ Ca\left(\sigma, \tau \right)=\sum \limits_{SNP}\frac{\left|\tau (SNP)-\sigma (SNP)\left|\right.\right.}{\tau (SNP)+\sigma (SNP)} $$

where SNP varies across all SNPs. Essentially, in calculating the difference between the two ranked lists, this weights variations in the lower portion of the list less than those in the top. Since the most important SNPs are those in the upper part of the ranked lists, a variation of the Canberra distance consists in focusing on top-*k* lists and measuring the distance between two ranked lists using a Canberra distance with a location parameter *k + 1* for a desired *k*. This, denoted by Ca^(*k + 1*)^, is defined by

$$ {Ca}^{\left(k+1\right)}\left(\sigma, \tau \right)=\sum \limits_{SNP}\frac{\left|\min \Big[\tau (SNP),k+1\left]-\min \right[\sigma (SNP),k+1\Big]\left|\right.\right.}{\begin{array}{l}\min \left[\tau (SNP),k+1\right]+\min \left[\sigma (SNP),k+1\right]\\ {}\end{array}}. $$

For each data set we computed all pairwise distances between the 25 approaches using the Canberra distances with location parameter *k + 1* for *k =* 100, 200, 500, 1000, 5000, 10,000, 50,000, 100,000 as well as using the basic Canberra distance (note that the basic Canberra distance Ca is equivalent to Canberra with location parameter *N + 1* where *N* is the total number of SNPs in the data set). This was done with an in-house python script (available upon request) leveraging the pandas [18], numpy [19] and scipy libraries [20].

Hierarchical clusterings from the Canberra-based distance metrics were generated in R with ‘hclust’ (linkage = “average”) and the R ‘clue’ library (https://cran.r-project.org/package=clue; [21]) was used to calculate pairwise clustering agreements and to generate consensus clusterings (with method set to “euclidean”). The R function ‘cutree’ was used to extract the specified number of clusters from the hierarchical clusterings. Clustering heat maps leveraged the above python libraries and the seaborn (https://pypi.org/project/seaborn/) and matplotlib [22] libraries. For these, dissimilarity values were first normalized by the maximum dissimilarity.

## Results

For each data set, we generated hierarchical clusterings of the 25 univariate approaches from each of the nine Canberra-based distance metrics Ca^(*k + 1*)^ for *k =* 100, 200, 500, 1000, 5000, 10,000, 50,000, 100,000 and Ca. For each pair of distance metrics, we then compared the resulting clusterings using the cophenetic correlation coefficient, which is the Pearson correlation coefficient between the ultrametric distances corresponding to each of the two clusterings. For clusterings of the approaches applied to the GENEVA data set, agreement was very strong (> 0.97) for all pairs of clusterings from metrics with *k ≤* 10,000*,* dropping to around 0.5 for pairs of clusterings involving Ca^(*50,001*)^. For clusterings of the approaches applied to the BPC3 data set, agreement was very strong (> 0.97) for all pairs of clusterings from metrics with *k* ≤ 1000, dropping to around 0.3 for pairs of clusterings involving Ca^(5001)^. Based on this, we used *k*-thresholds of 10,000 and 1000 respectively for consensus clusterings of ranked lists from GENEVA and BCP3 below. We expect different numbers of trait-associated SNPs for T2D and breast cancer. To date there are close to ~ 490 distinct loci that have been associated to T2D [23–25] and ~ 170 distinct loci that have been associated to overall breast cancer [26]. Thus, it is not surprising that noise SNPs start dominating the method clusterings based on results for the BPC3 data set at smaller *k* values than for GENEVA.

**Fig. 1:**
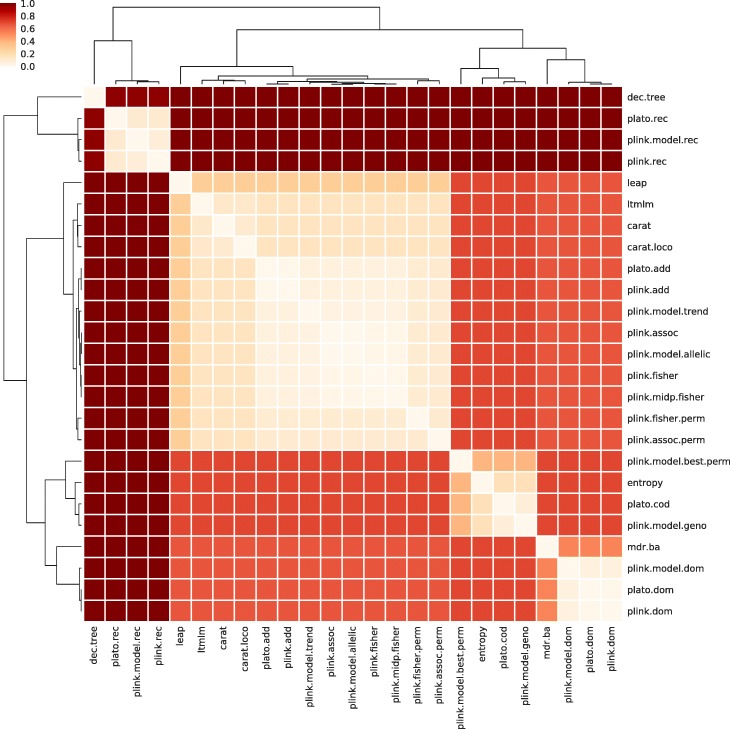
Consensus of the six Canberra-based distance metrics clusterings from Ca^(*k + 1*)^ for *k* = 100, 200, 500, 1000, 5000, 10,000 for the 25 univariate analysis approaches applied to the GENEVA data set. Heatmap cells indicate dissimilarity (the darker the more dissimilar) normalized to the max dissimilarity

The consensus of the six clusterings from Ca^(*k + 1*)^ for *k* = 100, 200, 500, 1000, 5000, 10,000 for the 25 univariate analysis approaches applied to the GENEVA data set is presented in Fig. 1 (the consensus clustering for the BPC3 data set is provided in Additional file 1). The PLINK logistic regression with additive encoding (abbreviated plink.add) yielded top ranked SNP lists very close to those from PLATO with additive encoding (plato.add), which was expected as these are two implementations of the same type of analysis. The same holds for PLINK logistic regression and PLATO with dominant encoding (plink.dom and plato.dom) and with recessive encodings (plink.rec and plato.rec). The PLINK recessive model (plink.model.rec) also clustered close to the PLINK logistic regression and PLATO with recessive encoding. Similarly, the PLINK model dominant (plink.model.dom) clustered close to the PLINK logistic regression and PLATO with dominant encoding in the clustering from GENEVA, whereas in the clustering from BPC3 the similarity was much weaker. The various PLINK basic association tests generated similar rankings of top SNPs, also similar to the PLINK model allelic and the PLINK model trend test. The choice of global GRM versus applying a LOCO approach with CARAT did not affect the resulting top rankings very much. Finally, the ranked lists from entropy were similar to those from the PLINK genotypic model (plink.model.geno). All of these are general trends that we observed in the clusterings from both data sets. However, at a finer resolution, we expect that clusterings of approaches based on analysis results may well differ from one data set to another, even when looking at the same trait. This is because some methods were developed with specific issues in mind (e.g. to address the presence of hidden population structure, strong ascertainment bias, etc.), even though they are typically applicable also when those issues do not occur in a data set. Thus, depending on whether or not a particular issue which a method was designed to address occurs in a given data set, the results from that method may be less or more similar to those from other methods.

**Table 1:**
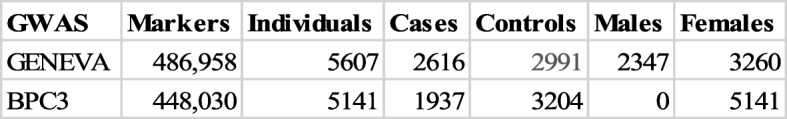
Number of markers and individuals in each data set after pre-processing, with breakdown of individuals by phenotype and sex

**Table 2:**
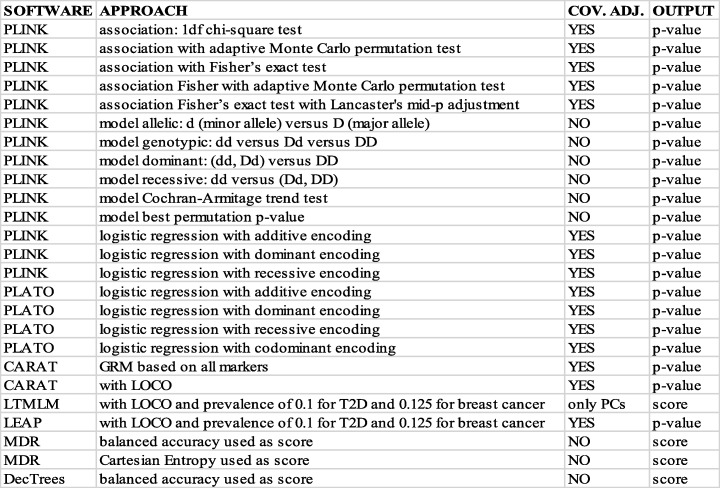
Univariate analysis approaches used in this work

COV. ADJ. indicates whether the method allowed for covariate adjustments

From the consensus clusterings, it appears that cutting the tree at 8 clusters for GENEVA and 9 clusters for BPC3 strikes a good balance towards eliminating redundancies without sacrificing diversity. For GENEVA, we could pick the following representative approaches from the resulting 8 distinct clusters (nicknames used in the figures are indicated in parentheses): PLINK logistic regression with additive (plink.add), dominant (plink.dom), and recessive (plink.rec) encodings, LEAP (leap), MDR (mdr.ba), entropy (entropy), PLINK model with permutation (plink.model.best.perm), and Decision Trees (dec.tree). For BPC3, we could choose as representative these same 8, plus PLINK model dominant (plink.model.dom).

For each GWAS data set, we term *top-k union* the collection of all SNPs ranking in the top *k* in at least one of the selected representative approaches. The number Ʃ_*k*_ of SNPs in the top-*k* union is termed *union number* [17]. If the top *k* SNPs in all representative lists were the same (complete stability), then Ʃ_*k*_ would equal *k*, whereas if the top *k* SNPs in the representative lists were pairwise disjoint (complete instability) then Ʃ_*k*_ would equal *8 k* in GENEVA and *9 k* in BPC3. Table 3 reports the union numbers for the values of *k* used in the consensus clusterings. For GENEVA the union numbers vary from about 40 to 48% of complete instability, and for BPC3 they are around 55% of complete instability, indicating that there is a considerable amount of diversity in the top results as the approach used varies across the representatives we selected.

Following terminology from [17], for a given *k*, the *extraction number* for a SNP in the top-*k* union is the number of representative methods for which the SNP ranks in the top *k* and its *average position number* denotes the average rank across the methods for which the SNP is in the top *k.* High extraction numbers and low average positions indicate SNPs that are in the top *k* for multiple methods. We ran our univariate analyses on all genotyped SNPs (after QC). To better assess the independent signals in the top-*k* unions, we first grouped SNPs into haplotype blocks (using PLINK --blocks) and then selected a representative for each block, by taking the most ‘stable’ representative (in terms of extraction number, followed by average position). We call the set of such representatives the *pruned top-k union*. Table 4 has the statistics on such sets for various values of *k*.

**Fig. 2:**
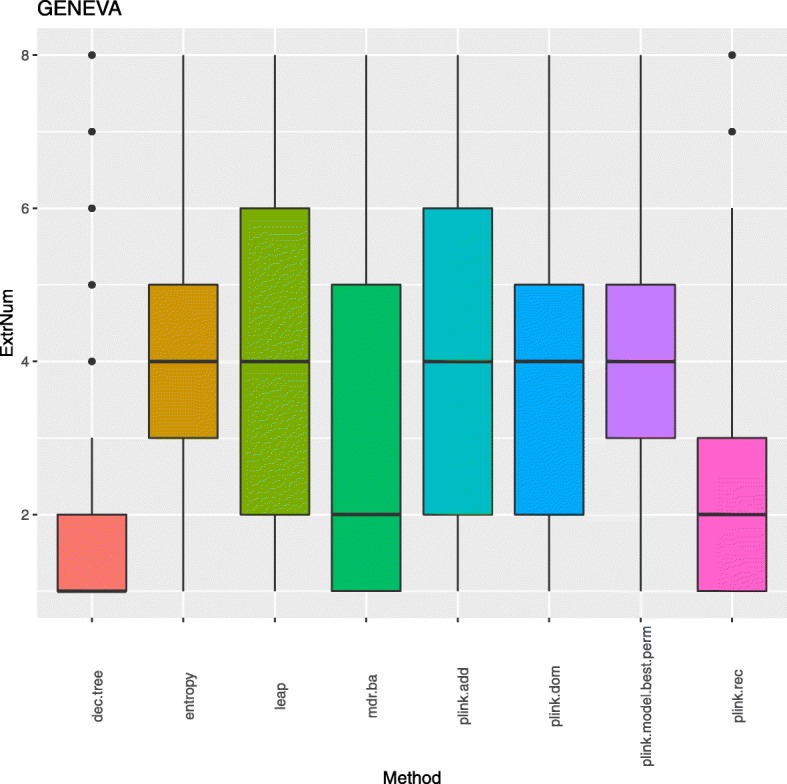
Boxplots for the extraction numbers of the SNPs in the GENEVA pruned top-*1000* union for the 8 representative approaches

As mentioned earlier, about ~ 490 loci and ~ 170 respectively have been reported as associated to T2D and overall breast cancer to date. Since the most common approach used in the literature to identify such loci is logistic regression with additive encoding, we have taken a closer look at the list of SNPs in the pruned top-*1000* union for GENEVA and the pruned top-*200* union for BPC3, given that the number of independent signals in these sets which are in the top *k* for plink.add are the closest (see Table 4) to the numbers of trait associated loci reported to date. To explore how much each approach may contribute to the diversity of independent top *k* signals for these two respective values of *k*, we looked at the distributions of extraction numbers by approach for the SNPs in the pruned top-*1000* union for GENEVA and in the pruned top-*200* union for BPC3. Figure 2 illustrates boxplots of these distributions for the GENEVA data. This indicates that the top independent signals identified by logistic regression with additive encoding and LEAP tend to also be picked up by other approaches with a median of 4 approaches and an interquartile range of 2–6. The top independent signals identified by logistic regression with dominant encoding, plink.model.best.perm, and entropy also tend to be identified by a median of 4 approaches, but with smaller 3rd quartile and, in the case of plink.model.best.perm and entropy, a higher 1st quartile. The top independent signals identified by MDR and logistic regression with recessive encoding tend to be identified only by a median of another approach, with a smaller 3rd quartile for the latter. Finally, the top independent signals identified by decision trees tend be solely identified by this approach. The corresponding results for BPC3 are discussed in Additional file 2.

**Table 3:**
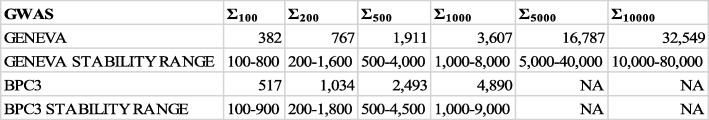
Union numbers Ʃ_*k*_ for the different values of *k* employed in the consensus clustering for the indicated data set, based on the ranked SNP lists for the representative approaches

Stability ranges are also indicated

**Table 4:**
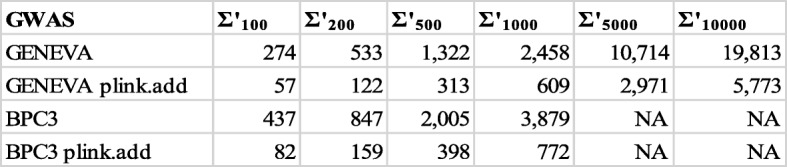
The rows GENEVA and BPC3 report the numbers Ʃ’_*k*_ of SNPs in the pruned top-*k* unions for the different values of *k* employed in the consensus clustering for the indicated data set

The rows marked by plink.add report the number of independent signals in the top *k* for the plink.add approach

**Fig. 3:**
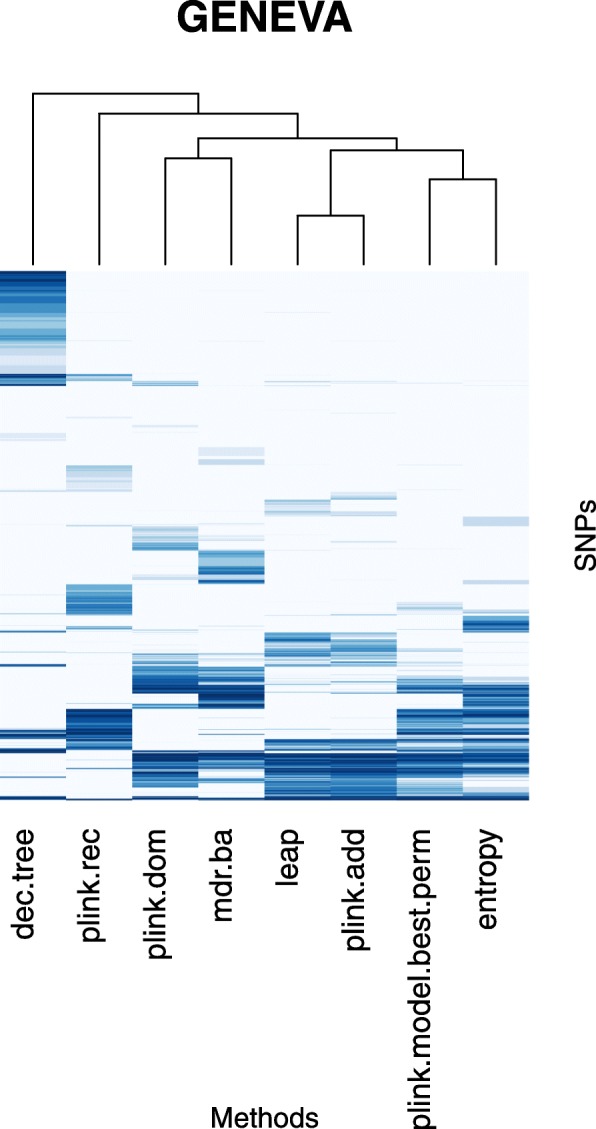
Hierarchical heatmap of the GENEVA pruned top *1000-*union across the 8 approaches. Darker cells correspond to better rankings; white cells indicate SNPs not in the top 1000 for that approach

Figure 3 displays a hierarchical heatmap of the pruned top *1000-*union for the GENEVA data set. Additional file 3 contains the details about this list. Given the limited sample size, as compared to the typical sample size from consortia data, GENEVA per se has limited power, but established T2D signals are enriched in this list. Interestingly, whereas some of these are being identified by the most popular approaches (such as logistic regression with additive encoding), others are only identified in this data set by one or two of the least commonly used approaches. Moreover, all the established signals in the pruned top *1000-*union identified by logistic regression with additive encoding, are also identified by at least one of the other approaches. As examples, rs7901695 which is in high LD (r^2^~0.87, from http://biostats.usc.edu/software, using all European populations) with an established T2D locus (rs7903146 ‘at *TCF7L2*’), ranks very highly in all of the methods. Similarly, rs1020731 which is a proxy for an established sentinel ‘at *RBMS1*’ (rs3772071), ranks very highly in all methods but decision trees. On the other hand, rs2249105 (an established sentinel ‘at *CEP68*’), rs1009358 (a proxy for another established sentinel ‘at *CEP68*’), rs1035061 (a proxy for an established sentinel ‘at *BPTF*’), and rs1495381 (a proxy for an established sentinel ‘at *TSPAN8*’) are identified among the top 1000 only with logistic regression with recessive encoding, whereas rs3094187 (in the same haplotype block as the established sentinel rs3130501 at ‘*POU5F1-TCF19*’) is only among the top 1000 of logistic regression with dominant encoding. As another example, rs2076578 (in the same haplotype block as the established sentinel rs5758223 ‘at *EP300*’) and rs3843467 (a proxy for an established sentinel ‘at *ANKRD55*’) are among the top 1000 only with decision trees. Finally, rs11708067 (an established sentinel ‘at *ADCY5*’) is only among the top 1000 when using MDR and the corresponding model predicts as risk genotypes the two homozygous genotypes. Besides these established loci, other interesting signals appear in the pruned top-*1000* union as identified only by less frequently used methods, as the following examples illustrate. rs132539 is only identified in the top 1000 for decision trees and it is reported as an eQTL in pancreas for *XBP1* (from GTEx v7, https://www.gtexportal.org), a gene whose beta-cell ablation in mouse results in decreased insulin secretion [27]. rs10865895 is only identified in the top 1000 for logistic regression with recessive encoding and it too is an eQTL in pancreas for *ENTPD3* (from GTEx), a gene encoding the NTPDase3 enzyme which regulates glucose-induced insulin secretion [28]. rs972297 is only identified in the top 1000 by MDR and, based on its annotation in HaploReg v 4.1 [29], it resides within enhancer regions in various T2D relevant tissues, such as pancreatic islet, adipose, liver, and skeletal muscle. In addition, based on Chip-seq in the HepG2 cell line (a liver model) it is within a binding region for *CEBPB*, a transcription factor up-regulated in the diabetic liver [30]. rs10519678 is only identified in the top 1000 by LEAP and is in high LD (r^2^ = 1) with rs11100782, a SNP within an enhancer region in pancreatic islet (HaploReg v4.1), which has been detected as a chromatin accessibility QTL (caQTL) in this tissue [31]. Discussion of the pruned top *200*-union for the BPC3 data set is provided in Additional file 2.

## Discussion

**Fig. 4:**
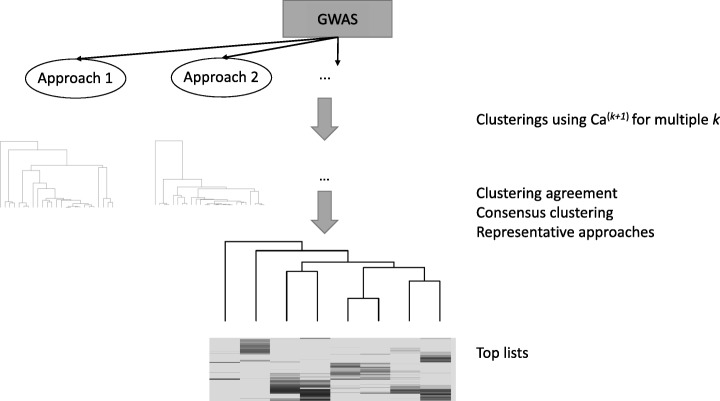
Workflow for multi-approach analysis strategy. Different applicable approaches are each run on the given GWAS. Clusterings of these approaches are then generated utilizing Canberra based metrics of dissimilarity, with location parameter *k*, between the resulting ranked SNP lists. Clustering agreement is then assessed to select the values of *k* on which a consensus clustering is based. From the consensus clustering, a subset of representative approaches is selected and top SNP lists are generated for these. Depending on scope of follow-up, size of the GWAS, and approaches employed, these lists could be based on a top cutoff or on significant *p*-values, after multiple testing corrections

Using two GWAS data sets for different binary traits (T2D and breast cancer), we have examined the variability in univariate analysis results across several applicable methods implemented in different software packages, both of a statistical and of a machine learning nature. Figure 4 outlines the workflow that we followed. We have utilized Canberra-based distance metrics on the resulting ranked lists of genotyped SNPs to generate hierarchical clusterings of the univariate approaches. As the choice of the optimal length of ranked lists for computing Canberra distance is unknown, we have performed hierarchical clusterings for selected lengths, assessed clustering agreements, and generated a consensus clustering up to the length after which the agreements started to decrease. Based on the clustering results, we have extracted a list of representative approaches to capture different slices of the sets of trait-associated variants. Examination of the top-most ranked SNPs across these approaches highlighted on the one hand that, on these data sets, the methods most typically used in GWAS analyses tended to identify at the top SNPs which were also detected by other approaches. On the other hand, some of the approaches, like logistic regression with recessive encoding or decision trees, presented several signals at the top that were solely detected by a single method and that are of interest for the trait, based on literature and eQTL evidence. It is conceivable that by using only a single approach, such as logistic regression with an additive encoding (the most popular approach in the literature), there could be a higher false negative rate and important biological signals may be missed. Moreover, when we examined established, replicated T2D loci from large consortia, we noticed that in the (relatively small) GENEVA data set, some signals were only detected at the top ranks in our analyses for one or a few of the less commonly used approaches.

The scope of this work was not to survey and compare all available approaches for binary GWAS univariate analyses. For a systematic assessment of the strength and weaknesses of different approaches, one would need to work with simulated GWAS data sets spanning a variety of situations (e.g. cryptic relatedness, ascertainment bias, etc.). More work in the area of GWAS simulation is needed to get to the point of generating data sets covering all subtle scenarios needed for a thorough comparison of methods. Also, our intent is not to recommend a set of specific methods that should be used in all cases, as this would not be appropriate since the methods applicable to a given data set depend on the assumptions one can make about that dataset (for our case studies, we selected a collection of applicable methods and varied the encoding choices as well). Thus, we are not suggesting that the list of approaches to apply should consist of the representative approaches that we used for the specific GWAS in this work. For example, CARAT, which in our data sets clustered close to the PLINK logistic regression tests with additive encoding, may cluster differently in a data set where unknown population structure is impactful. Moreover, for certain data sets, it may not be appropriate to run PLINK tests that do not keep into account unknown population structure and cryptic relatedness. What our work indicates instead is that, rather than fixing a choice of analyses for a given GWAS, it may be advisable to apply multiple applicable approaches, spanning different modeling and encoding assumptions. Clustering of approaches based on resulting ranked lists can then be used to reduce redundancies (hence the multiple testing burden) and select a set of representative approaches. Then the collection of results from these approaches could provide a larger pool of candidates for subsequent follow-up.

Application of multiple approaches brings up the issue of multiple testing when the aim is to identify statistically significant hits. Because the GWAS we had available for this study consist of relatively small cohorts, this was not our aim in this work. Rather, our aim was to compare the top signals across methods to explore how these methods differed on our data. When focusing on top *k* lists, we selected *k* based on current knowledge about the numbers of loci identified by large consortia for the given traits. When larger cohorts are available for a trait, one can incorporate meta-analyses and multiple testing corrections (at least for methods yielding *p*-values) across the approaches. Moreover, for some of the methods which typically yield scores instead of p-values (e.g. MDR), it is possible to compute permutation p-values which could be used instead, if one wanted to apply multiple testing adjustments to identify significant candidates. In order to increase power for the study of complex traits, it is often common for investigators to join large consortia, within which each group carries out a GWAS for that trait. Design and analysis criteria are typically agreed upon and each group analyzes their study according to those criteria. Then individual genotypes are kept confidential, but the generated summary statistics are shared to enable meta-analyses. This design is still compatible with a workflow which employs multiple approaches. Indeed, in the first phase, each group could apply a battery of applicable approaches to their GWAS and produce a distance matrix between approaches (the latter could be derived from a consensus clustering across different Canberra based similarity metrics). The distance matrix from a GWAS bears no information about SNP genotypes and can be shared. Distance matrices can be used to generate approach clusterings for each GWAS and pairwise agreements between the clusterings from the different GWAS can then be assessed and a consensus clustering can be derived. The latter would indicate which representative approaches to use across the consortium. Then each group could simply share the summary statistics for these representatives. For each representative approach, the summary statistics could then be combined through meta-analyses to generate a list of candidate SNPs. Note that, at this stage, for each representative approach, one could select the threshold for the top candidates as appropriate, e.g. accounting for multiple testing in the case of *p*-values, and the union of the final top candidates across approaches would be the final output.

As a final remark, in this work we have focused on binary traits, based on the data sets to which we had full access. However, the same type of recommended workflow can be applied to the case of quantitative traits, as long as the chosen approaches are among those designed for quantitative traits. Similarly, here we only used genotyped SNPs so we could employ a larger number of methods. In the presence of imputed SNPs, it is advisable to employ methods specifically designed so to exploit posterior genotype probabilities [6]. Also we were interested in looking at pruned top lists, so having additional imputed SNPs in LD would have been an unnecessary redundancy. But, when the focus is on the identification of significant signals and imputation is performed, the same workflow can still be used, with care in selecting methods which are designed to analyze data which include imputed genotypes.

## Additional files

Additional file 1: BPC3 consensus clustering. Consensus of the four Canberra based distance metrics clusterings from Ca^(*k + 1*)^ for *k* = 100, 200, 500, 1000) of 24 univariate analysis approaches applied to the BPC3 data set (the Decision Tree approach is not displayed to improve visualization, since it was highly dissimilar from all others). Heatmap cells indicate dissimilarity (the darker the more dissimilar) normalized to the max dissimilarity. (PDF 21 kb)

Additional file 2: Additional Results. Discussion of results relative to the BPC3 data set (Additional files 4, 5 and 6). (DOCX 18 kb)

Additional file 3: GENEVA pruned top-1000 union. GENEVA pruned top-1000 union, sorted by decreasing extraction number followed by increasing average position. For each SNP in this list, its extraction number and average position in the ranking are indicated. For each approach, a 0 indicates that the SNP was not in the top 1000, a non-zero value occurs if the SNP was in the top 1000 and indicates how well the SNP ranks with 1000 being the best ranking and 1 the worst. (XLSX 140 kb)

Additional file 4: BPC3 extraction numbers boxplots. Boxplots for the extraction numbers of the SNPs in the BPC3 pruned top-200 union for the 9 representative approaches. (PDF 5 kb)

Additional file 5: BPC3 pruned top 200-union heatmap. Hierarchical heatmap of the BPC3 pruned top 200-union across the 9 approaches. Darker cells correspond to better rankings; white cells indicate SNPs not in the top 200 for that approach. (PDF 29 kb)

Additional file 6: BPC3 pruned top 200-union. BPC3 pruned top-200 union, sorted by decreasing extraction number followed by increasing average position. For each SNP in this list, its extraction number and average position in the ranking are indicated. For each approach, a 0 indicates that the SNP was not in the top 200, a non-zero value occurs if the SNP was in the top 200 and indicates how well the SNP ranks with 200 being the best ranking and 1 the worst. (XLSX 56 kb)

## Data Availability

The GWAS data sets used for this work are available, upon application, from the database of Genotypes and Phenotypes [10] under the indicated accessions. These are: • The GENEVA Genes and Environment Initiatives in T2D, available under phs000091.v2.p1 (https://www.ncbi.nlm.nih.gov/projects/gap/cgi-bin/study.cgi?study_id=phs000091.v2.p1). • The breast cancer component of the Breast and Prostate Cancer Cohort Consortium (BPC3) GWAS of Aggressive Prostate Cancer and ER- Breast Cancer, available under phs000812.v1.p1 (https://www.ncbi.nlm.nih.gov/projects/gap/cgi-bin/study.cgi?study_id=phs000812.v1.p1). The custom scripts used for this work are available upon request.

## Abbreviations

CARAT: Case-control retrospective association test
GRM: Genetic relationship matrix
GWAS: Genome wide association study
LD: Linkage disequilibrium
LEAP: Liability estimator as a phenotype
LOCO: Leave-One-Chromosome-Out
LTMLM: Liability-Threshold Mixed Linear Model
MDR: Multifactor dimensionality reduction
PC: Principal Component
SNP: Single nucleotide polymorphism
T2D: Type 2 diabetes
