# Supplementary figures and images for: Exploration of a diversity of computational and statistical measures of association for genome-wide genetic studies
Source: BioData Min. 2019 Jul 9;12:14. doi: 10.1186/s13040-019-0201-4 (PMC6617598; doi:10.1186/s13040-019-0201-4)

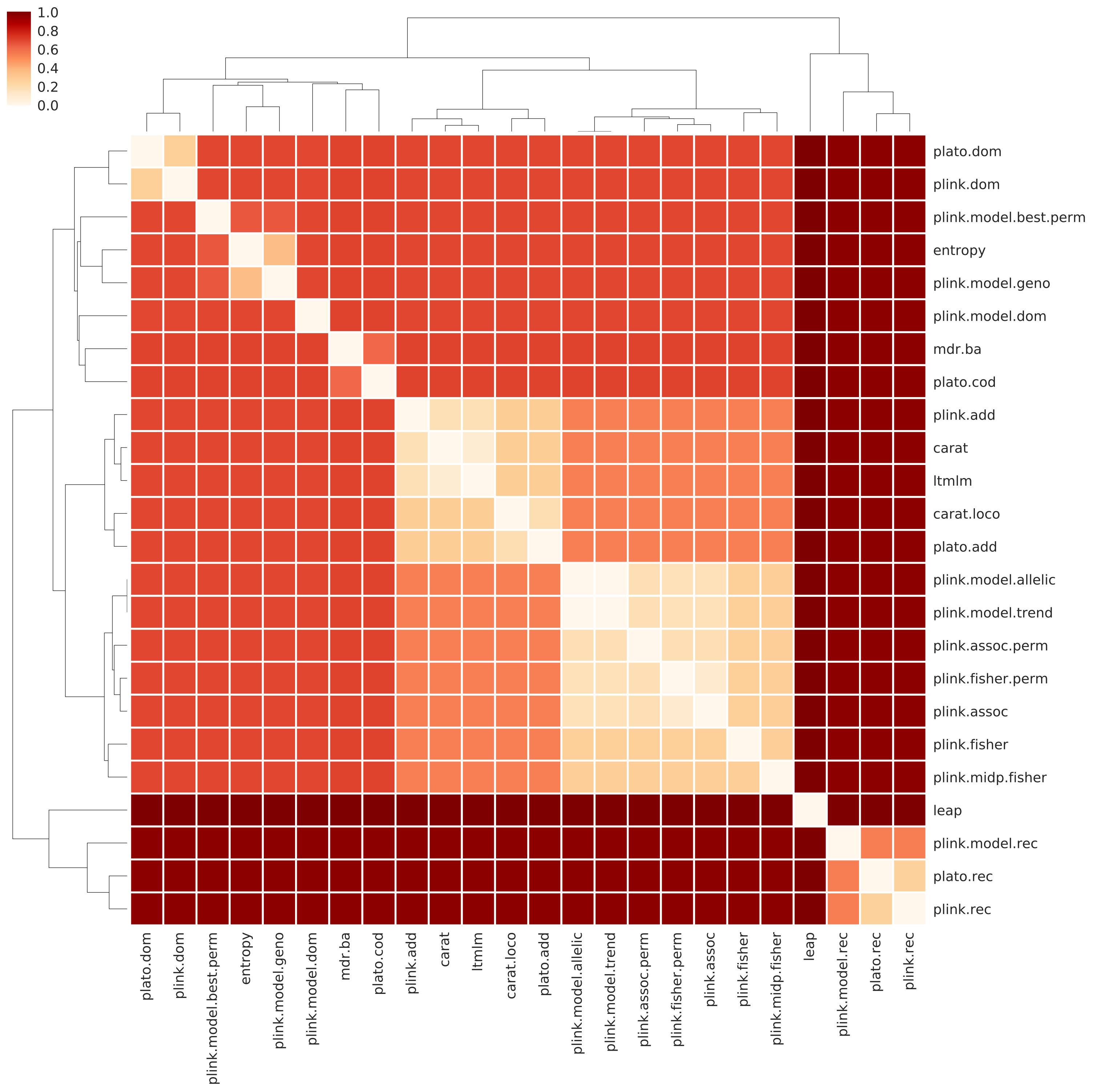

Supplement: Supplementary file 1 — BPC3 consensus clustering. Consensus of the four Canberra based distance metrics clusterings from Ca(k + 1) for k = 100, 200, 500, 1000) of 24 univariate analysis approaches applied to the BPC3 data set (the Decision Tree approach is not displayed to improve visualization, since it was highly dissimilar from all others). Heatmap cells indicate dissimilarity (the darker the more dissimilar) normalized to the max dissimilarity. (PDF 21 kb) [file 13040_2019_201_MOESM1_ESM.pdf]

BPC3

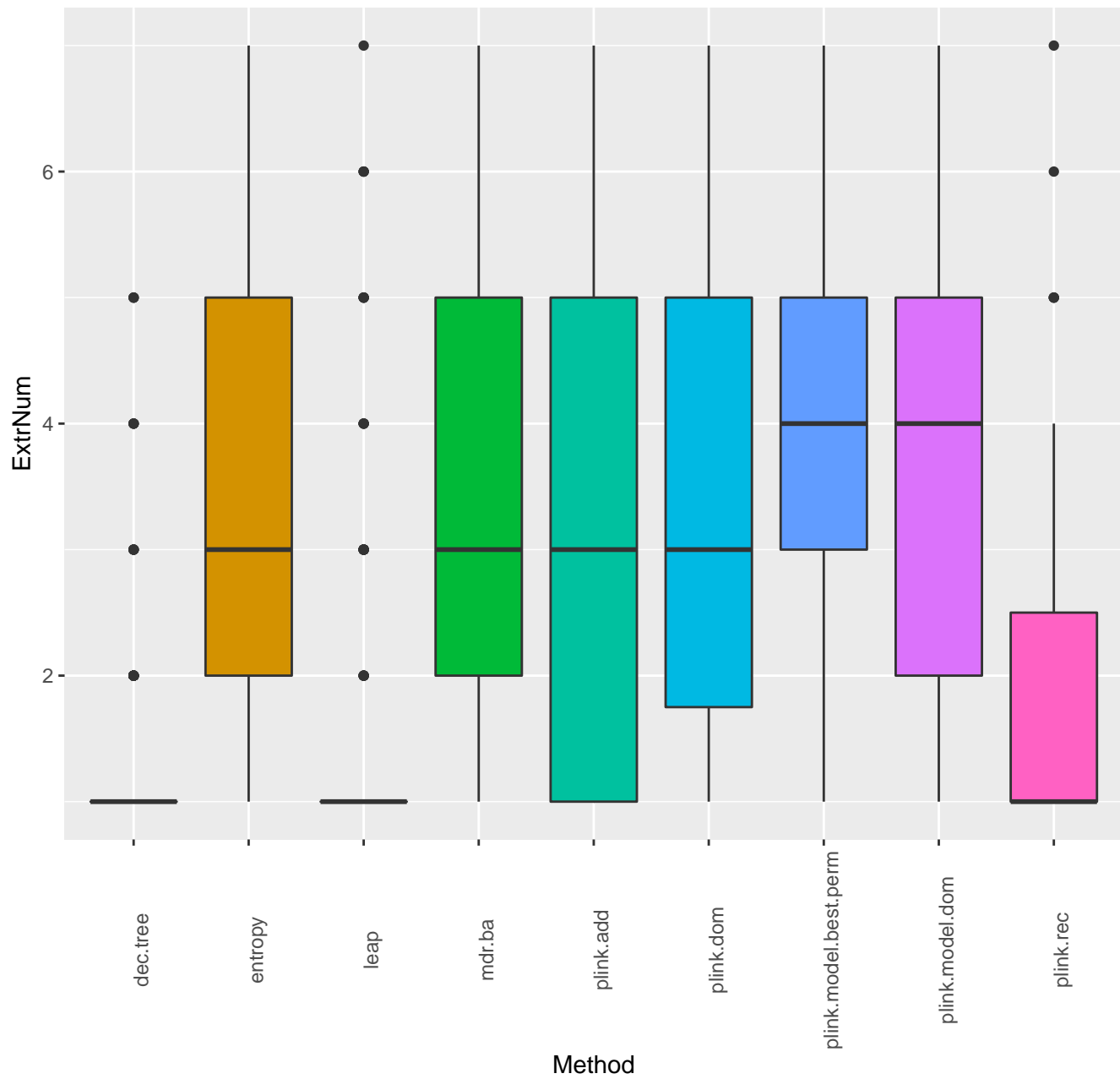

Supplement: Supplementary file 4 — BPC3 extraction numbers boxplots. Boxplots for the extraction numbers of the SNPs in the BPC3 pruned top-200 union for the 9 representative approaches. (PDF 5 kb) [file 13040_2019_201_MOESM4_ESM.pdf]

# BPC3

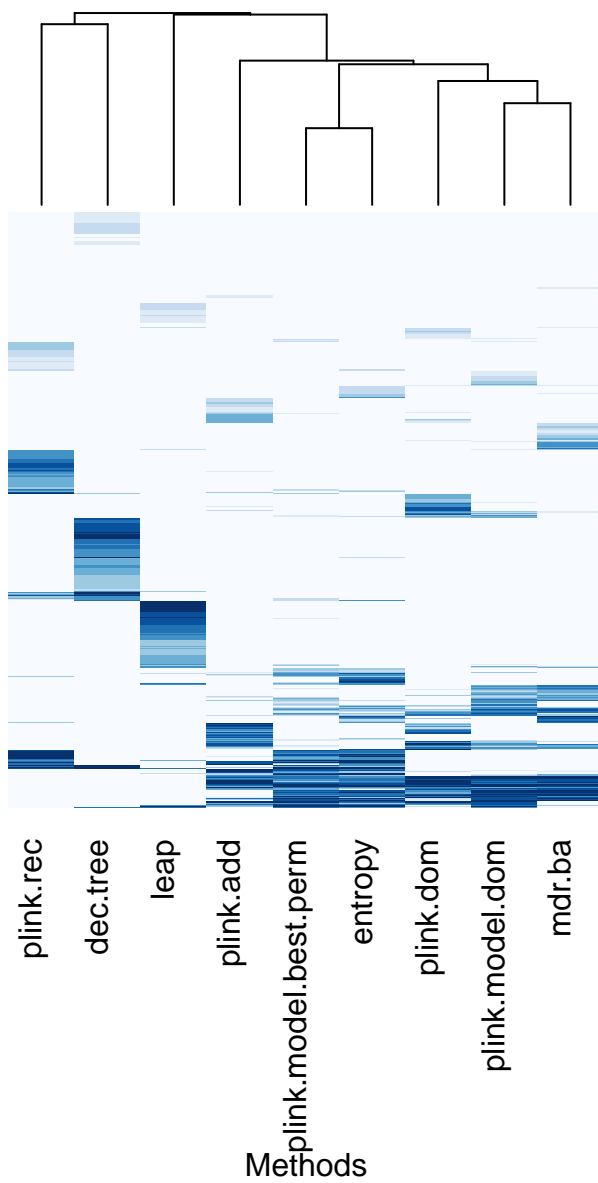

Supplement: Supplementary file 5 — BPC3 pruned top 200-union heatmap. Hierarchical heatmap of the BPC3 pruned top 200-union across the 9 approaches. Darker cells correspond to better rankings; white cells indicate SNPs not in the top 200 for that approach. (PDF 29 kb) [file 13040_2019_201_MOESM5_ESM.pdf]
