# Supplementary material for: Exploration of a diversity of computational and statistical measures of association for genome-wide genetic studies
Source: BioData Min. 2019 Jul 9;12:14. doi: 10.1186/s13040-019-0201-4 (PMC6617598; doi:10.1186/s13040-019-0201-4)
Supplement: Supplementary file 2 — Additional Results. Discussion of results relative to the BPC3 data set (Additional files 4, 5 and 6). (DOCX 18 kb) [file 13040_2019_201_MOESM2_ESM.docx]

**Additional Results**

Boxplots for the distributions of extraction numbers by approach for the SNPs in the pruned top-*200* union for BPC3 are shown in Additional File 4. The top independent signals identified by decision trees tend to be uniquely identified by this approach. For this data set, a similar situation occurs for the top independent signals identified by LEAP. The top independent signals identified by logistic regression with recessive encoding also have a median extraction number of 1, but a higher 3^rd^ quartile. MDR, entropy, and logistic regression with additive and dominant encodings have similar extraction number distributions, with a median of 3 methods identifying the top independent signals. Independent signals identified at the top by plink.model.best.perm and plink.model.dom tend to be identified by a median of 3 additional methods.

Additional File 5 displays a hierarchical heatmap of the pruned top-*200* union for the BCP3 data set and Additional File 6 contains the details about this list. Also in this case we have an enrichment of established overall breast cancer signals, albeit less strong than in GENEVA. This may in part be due to the fact this data set only comprises ER-negative cases. Indeed, two (rs17530068 and rs9383938) of the three established overall breast cancer signals detected in this list are proxies to sentinels further refined to ER-negative signals in [1]. In this case the detected established overall breast cancer signals are all among the top 200 identified by logistic regression with additive encoding, but all of them are also detected by at least one of the other 8 methods. However other interesting SNPs are being identified only by few less typical approaches as illustrated in the following examples. rs2276102 is a missense variant within *TMEM135*, a gene reported as expressed in breast cancer in the Human Protein Atlas (https://www.proteinatlas.org/; [2]) and reported as an additional potential driver of breast cancer [3]. This SNP appears in the top 200 for logistic regression with recessive encoding, entropy and plink.model.best.perm only. rs2347889 is in the top 200 only for LEAP; this SNP is an eQTL for *C1GALT1* in breast mammary tissue (from GTEx), a gene whose up-regulation has been reported to promote breast cancer cell growth [4]. The SNP rs7048389, in the top 200 only for decision trees, is also reported in GTEx as an eQTL in breast mammary tissue for *FANCG*, a gene with direct interactions to *BRCA2* [5]. rs4132466 appears in the top 200 only for PLINK logistic regression with dominant encoding and PLINK model dominant and is in high LD (r^2^=1) with rs62156667, which is within a region with enhancer marks in HMEC Mammary Epithelial Primary Cells and affects the binding motif for PU.1 (HaploReg), a transcription factor that has been shown to have associations with breast cancer survival ([6]). rs1572349 appears in the top 200 only with MDR is in high LD (r^2^>0.96) with rs6424686, which is within a region with enhancer marks in HMEC Mammary Epithelial Primary Cells and affects the binding motif of AhR (HaploReg), a target for breast cancer therapy [7].

**References**

[1] Lilyquist J, Ruddy KJ, Vachon CM, Couch FJ. Common Genetic Variation and Breast Cancer Risk - Past, Present, and Future. Cancer Epidemiology, Biomarkers & Prevention. 2018;27:380-394.

[2] Uhlen M, Zhang C, Lee S, Sjöstedt E, Fagerberg L, Bidkhori G, et al. A pathology atlas of the human cancer transcriptome. Science. 2017;357(6352):eaan2507.

[3] Natrajan R, Mackay A, Lambros MB, Weigelt B, Wilkerson PM, Manie E, et al. A whole-genome massively parallel sequencing analysis of BRCA1 mutant oestrogen receptor-negative and -positive breast cancers. J Pathol. 2012;227:29-41.

[4] Chou CH, Huang MJ, Chen CH, Shyu MK, Huang J, Hung JS, et al. Up-regulation of C1GALT1 promotes breast cancer cell growth through MUC1-C signaling pathway. Oncotarget. 2015;6:6123-6135.

[5] Hussain S, Witt E, Huber PA, Medhurst AL, Ashworth A, Mathew CG. Direct interaction of the Fanconi anaemia protein FANCG with BRCA2/FANCD1. Hum Mol Genet. 2003;12:2503-2510.

[6] Lin J, Liu W, Luan T, Yuan L, Jiang W, Cai H, Yuan W, Wang Y, Zhang Q, Wang L. High expression of PU.1 is associated with Her-2 and shorter survival in patients with breast cancer. Oncol Lett. 2017;14:8220-8226.

[7] Powell JB, Goode GD, Eltom SE. The Aryl Hydrocarbon Receptor: A Target for Breast Cancer Therapy. J Cancer Ther. 2013;4:1177-1186.
